# Low-grade serous epithelial ovarian cancer: a comprehensive review and update for radiologists

**DOI:** 10.1186/s13244-021-01004-7

**Published:** 2021-05-11

**Authors:** Sofia Amante, Filipa Santos, Teresa Margarida Cunha

**Affiliations:** 1 Department of Radiology, Hospital do Divino Espírito Santo de Ponta Delgada, Avenida D. Manuel I, 9500-370 Ponta Delgada, Azores, Portugal; 2 Department of Pathology, Instituto Português de Oncologia de Lisboa Francisco Gentil, Lisbon, Portugal; 3 Department of Radiology, Instituto Português de Oncologia de Lisboa Francisco Gentil, Lisbon, Portugal

**Keywords:** Low-grade serous carcinoma, Epithelial ovarian carcinoma, Computed tomography, Magnetic resonance imaging, Imaging

## Abstract

Low-grade serous carcinoma (LGSC) is an infrequent subtype of ovarian cancer, corresponding to 5% of epithelial neoplasms. This subtype of ovarian carcinoma characteristically has molecular features, pathogenesis, clinical behaviour, sensitivity to chemotherapy, and prognosis distinct to high-grade serous carcinoma (HGSC). Knowing the difference between LGSC and other ovarian serous tumours is vital to guide clinical management, which currently is only possible histologically. However, imaging can provide several clues that allow differentiating LGSC from other tumours and enable precise staging and follow-up of ovarian cancer treatment. Characteristically, LGSC appears as mixed lesions with variable papillary projections and solid components, usually in different proportions from those detected in serous borderline tumour and HGSC. Calcified extracellular bodies, known as psammoma bodies, are also a common feature of LGSC, frequently detectable within lymphadenopathies and metastases associated with this type of tumour. In addition, the characterisation of magnetic resonance imaging enhancement also plays an essential role in calculating the probability of malignancy of these lesions. As such, in this review, we discuss and update the distinct radiological modalities features and the clinicopathologic characteristics of LGSC to allow radiologists to be familiarised with them and to narrow the differential diagnosis when facing this type of tumour.

## Key points

- Low-grade serous carcinoma (LGSC) is a rare subtype of ovarian cancer.
- Imaging can provide several clues that suggest the diagnosis of LGSC.
- Psammoma bodies can occur within serous tumours or metastases, especially in LGSC.
- MRI enhancement patterns help to discriminate benign, borderline, and malignant ovarian tumours.
- Malignancy’s predictive models contribute to the early diagnosis of ovarian cancer.

## Introduction

Ovarian tumours are divided into epithelial neoplasms, mesenchymal neoplasms, sex cord-stromal tumours, and germ cell tumours [1, 2].

Epithelial neoplasms are the most frequent, accounting for 90–98% of ovarian tumours. According to the 2020 World Health Organization (WHO) Classification of Tumours, they are divided into serous tumours, which include high-grade serous carcinoma (HGSC) (70%) and low-grade serous carcinoma (LGSC) (5%); mucinous tumours (3–4%); endometrioid tumours (10%); clear cell tumours (10–12%); Brenner tumours (< 5%); and other carcinomas [2, 3].

LGSC and HGSC have different morphology, pathogenesis, associated molecular events, response to chemotherapy, and prognosis [1, 4–6].

LGSC is an invasive serous tumour presenting low-grade malignant features that is diagnosed at a young age (median age between 43 and 47), has an indolent clinical course, and is relatively chemoresistant [1, 4]. LGSC is also associated with longer progression-free survival and overall survival than HGSC [1].

The discrimination between HGSC and LGSC has a high impact on clinical management due to their diverse prognoses and treatment strategies. LGSC primary treatment is cytoreductive surgery, in contrast to HGSC, which is preferentially treated with chemotherapy and surgery [1].

Certain radiological features may provide important clues to the diagnosis of LGSC; however, the distinction between HGSC and LGSC is sometimes difficult (Table 1) [1, 4]. As such, radiologists must be aware of this entity and be familiarised with its radiological findings to optimise imaging protocols and provide adequate management and timely treatment to these patients.

**Table 1:** Radiological main characteristics of borderline, low-grade and high-grade ovarian serous tumours

	Serous borderline tumour	Low-grade serous tumour	High-grade serous tumour
Size	> 5 cm	> 5 cm	> 5 cm
Tumour architecture	Unilocular or multilocular cystic tumours with well-defined margins and papillary projections, seen in 67% of cases; walls/septa with ≤ 3 mm can occur	Multicystic lobulated tumours with solid components, papillary projections and thick walls/septa; calcifications are frequent; necrosis is rare	Mixed cystic-solid or totally solid tumours with irregular contours; areas of cystic change, haemorrhage or necrosis are frequent
Time-intensity curve	Type 2	Type 3	Type 3
Peritoneal lesions and lymph node involvement	30% may have non-invasive peritoneal implants and lymph node involvement	Delayed dissemination through peritoneal metastases is frequent, but is also seen through lymph nodes; calcified psammoma bodies are common	Typically present with diffuse peritoneal metastases and lymph node involvement
Ascites	43% of cases have ascites	Not frequently seen	Massive ascites is common

Table based on the literature review [1–3, 5–8, 14, 24, 27]

## Morphology and pathogenesis

**Fig. 1:**
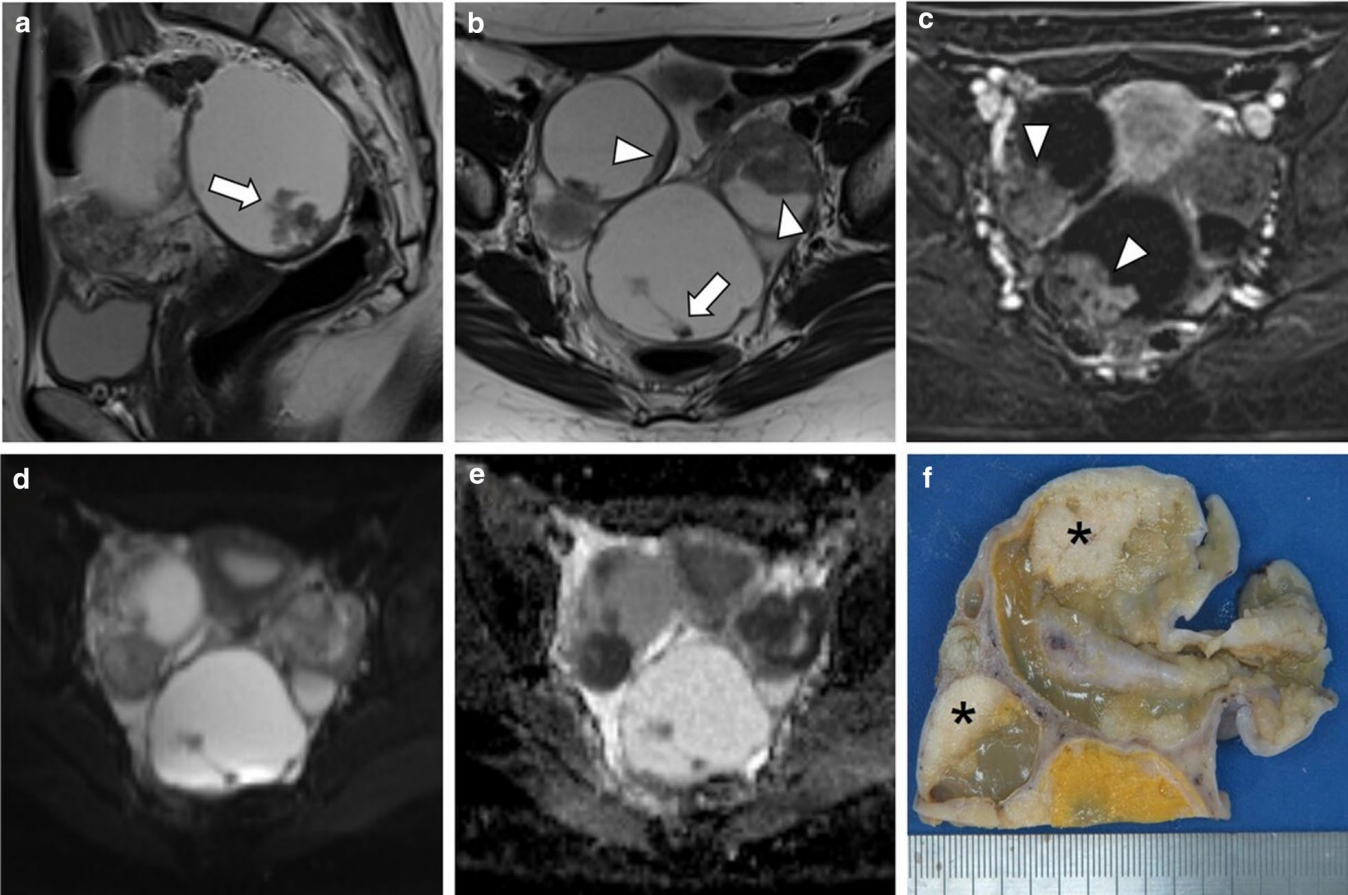
Bilateral LGSC associated with SBT and peritoneal metastases in a 33-year-old female. Sagittal and axial T2-weighted MR images (**a**, **b**) and axial post-contrast subtraction image (**c**) display a bilateral biloculated ovarian tumour, with papillary projections (arrows), that probably correspond to SBT component, and significant solid aspects (arrowheads) presumably attributable to LGSC component. Axial *b*-1000 s/mm^2^ diffusion-weighted image (**d**) and ADC map (**e**) show diffusion restriction in solid components. Gross section of surgical specimen of the left tumour (**f**). The ovary is replaced by a multicystic tumour with solid white nodules, corresponding to areas of LGSC (*). Small papillary projections were occasionally a component of SBT

Macroscopically, LGSC may present as bilateral adnexal tumours, often multicystic with nodular areas, excrescences, and papillary projections on the interior surface (Fig. 1). Some may be gritty due to the presence of calcifications [3].

**Fig. 2:**
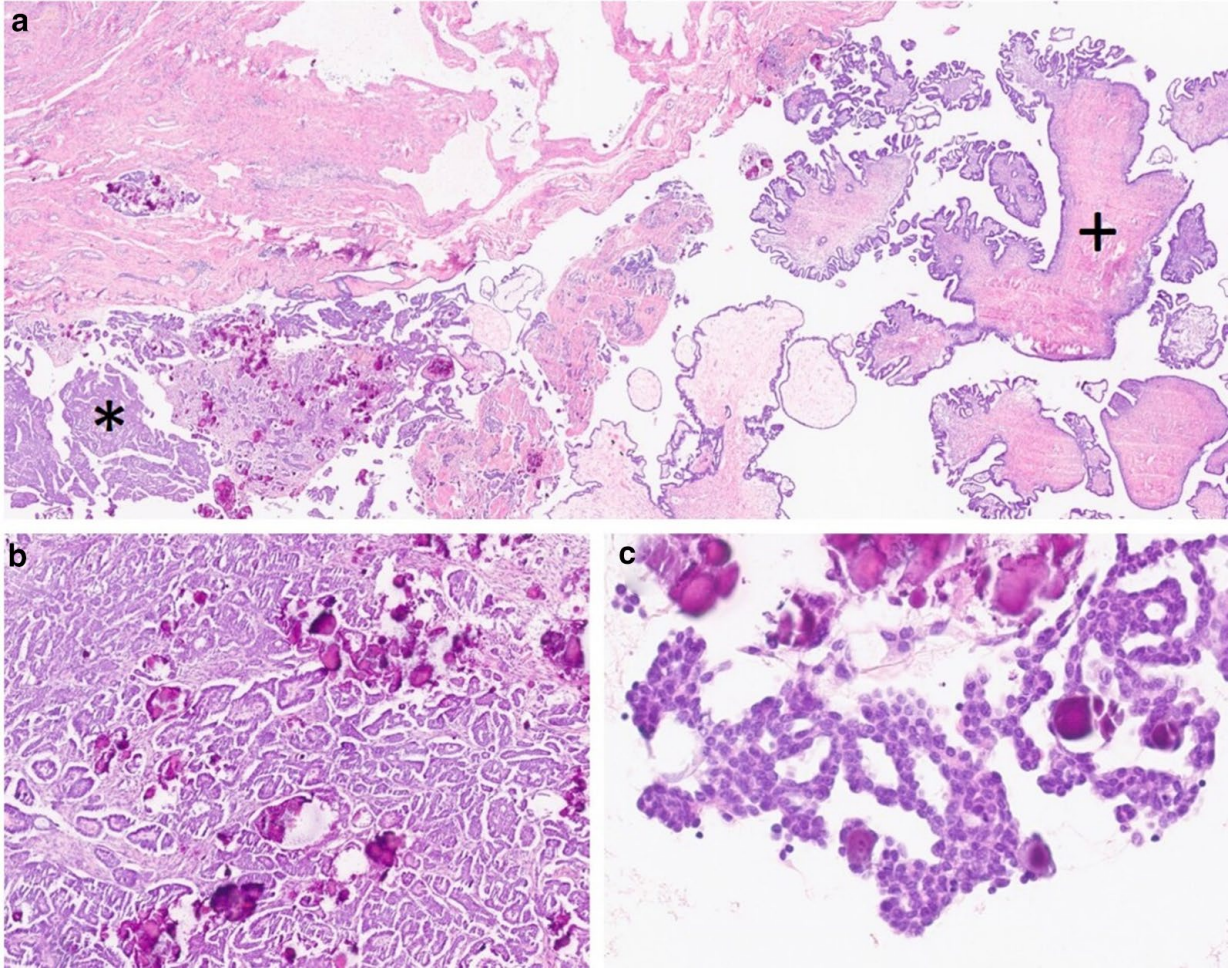
Microscopic examination of an LGSC associated with SBT. LGSC is present in the left inferior corner of the image (*) and an associated component of SBT is seen in the right side (+), H&E, × 10 (**a**). LGSC is composed of small nest, glands and papillae, immersed in a fibrous stroma, with abundant psammoma bodies, H&E, × 100 (**b**). In LGSC, papillae are covered with cells with bland appearance, uniform nuclei without significant atypia and no mitoses are seen; psammoma bodies are present; H&E, 200 × (**c**)

Microscopically, LGSC can have a diversity of morphological patterns, such as small nests, glands, papillae, or micropapillae. Frequently, there are micropapillae floating within clear spaces. There is mild to moderate atypia, with uniform nuclei. Compared to HGSC, LGSC has fewer mitoses (1–2 mitoses/mm^2^), and necrosis is usually absent. As in many other neoplasms with papillary growth, LGSC often has psammoma bodies. Approximately 60% of LGSC can occur with serous borderline tumour (SBT) at diagnosis [2–4] (Fig. 2).

Immunohistochemically, LGSCs are diffusely positive for CK7, PAX8, ER, and WT1, and p16 expression is patchy. In this carcinoma, as opposed to HGSC, p53 exhibits a wild-type pattern [3].

Whereas HGSC frequently arises de novo from tubal or ovarian surface epithelium, most LGSC develop in a stepwise fashion from serous cystadenomas, adenofibromas, and SBT [1, 2, 4–9].

LGSCs are often characterised by *KRAS*, *NRAS*, *BRAF*, *USP9X*, and *EIF1AX* mutations [1, 4, 6, 9]. Also, *KRAS* mutation may be related to tumour recurrence. Identification of these particular gene mutations may be useful for new clinical approaches and personalised treatments [2, 6, 9].

## Signs and symptoms

The signs and symptoms of LGSC are similar to other forms of ovarian tumours [10–12]. The patient can be asymptomatic or present with significant symptoms, mainly due to mass effect, such as early satiety, bloating, dyspnoea, urinary urgency, and pain. In advanced cases, it may course with pleural effusion and/or bowel obstruction [11, 12].

Although CA-125 serum levels tend to be higher in HGSC than in LGSC, CA-125 serum levels are used in the diagnosis and follow-up of LGSC, as in any other serous epithelial malignant ovarian tumour [10].

## Imaging findings of low-grade ovarian carcinoma

### Ultrasound

Ultrasound is typically performed as the first-line modality for characterising ovarian lesions [1, 6, 8].

Imaging features used to predict malignancy include thick irregular walls (> 3 mm), papillary projections, and solid echogenic nodules, with flow on colour Doppler [1].

These findings, integrated with additional clinical features, such as menopausal status and CA-125 level, allow risk stratification of adnexal lesions into likely malignant or benign by calculating the risk of malignancy index [1, 6, 8].

The International Ovarian Tumour Analysis (IOTA) group developed the Assessment of Different NEoplasias in the adneXa (ADNEX) model, which is a risk prediction model that involves three clinical and six ultrasound variables. This model can discriminate benign from malignant adnexal lesions with high sensitivity (97%) and specificity (71%) [1, 6]. However, about 25% of adnexal masses stay sonographically indeterminate even when evaluated by sonographic experts [13].

**Fig. 3:**
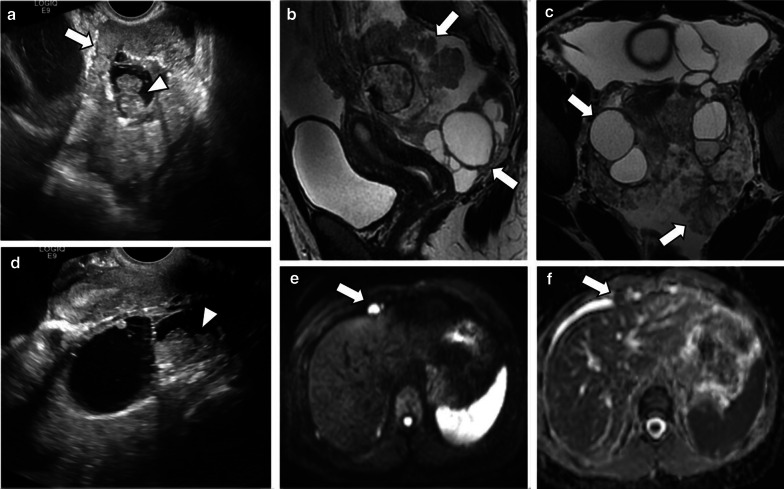
Bilateral LGSC with SBT and peritoneal metastases in a 25-year-old female. Ultrasound images (**a**, **d**) reveal a complex mixed tumour with a multinodular aspect involving adnexal structures (arrow). There is fallopian tubes' dilatation with solid echogenic vegetations inside (arrowheads). Sagittal and axial T2-weighted MR images (**b**, **c**) demonstrate a bilateral adnexal mixed tumour, with an arborescent growing on the surface of both ovaries (arrows) associated with severe ascites. Axial *b*-1000 s/mm^2^ diffusion-weighted image (**e**) and ADC map (**f**) show diffusion restriction in a perihepatic peritoneal metastasis (arrows)

It is known that the number of papillary projections and solid components increases from SBT to LGSC and to HGSC [8]. On ultrasound, LGSC usually appears as a multilocular cystic lesion with a higher number of solid components when comparing to SBT and with a lower number of solid components when compared to HGSC [1, 8, 10, 14] (Fig. 3). Calcifications corresponding to psammoma bodies are common in LGSCs and can be identified on ultrasound [3, 8]. On the other hand, HGSC appears more frequently as a non-papillary solid mass with areas of cystic change, necrosis, and/or haemorrhage [1, 10].

Doppler ultrasound may also be useful since HGSC tends to be more vascularised than LGSC and SBT [8]. In elastography, LGSC is usually stiffer and less elastic than an HGSC. This fact can be explained by the frequent presence of necrosis in HGSC [1, 10].

### Computed tomography

Contrast-enhanced CT is the current imaging modality of choice for ovarian cancer staging and for treatment follow-up [1, 2, 13, 15]. It allows the detection of lymphadenopathy and peritoneal metastases with high diagnostic accuracy (89%) [1, 15, 16].

The use of oral contrast is generally recommended to detect adnexal lesions and is also useful to distinguish peritoneal metastases from the fluid-filled bowel [1, 2, 16]. Oral contrast is especially necessary in women with low body mass index or in premenopause, in whom ovaries might be difficult to detect [2]. Generally, 1.5 L of diluted contrast or water is administered an hour before the study [2, 13].

Nevertheless, the sensitivity of CT to detect peritoneal metastases depends on their size, and is low for metastases smaller than 1 cm (25–50%) [1, 2, 15].

The use of intravenous contrast allows optimal characterisation of adnexal lesions architecture and identification of pelvic vascular structures. Solid components and papillary projections should be assessed on the venous phase (70–90 s) as they may be missed in the early phase [1, 13].

**Fig. 4:**
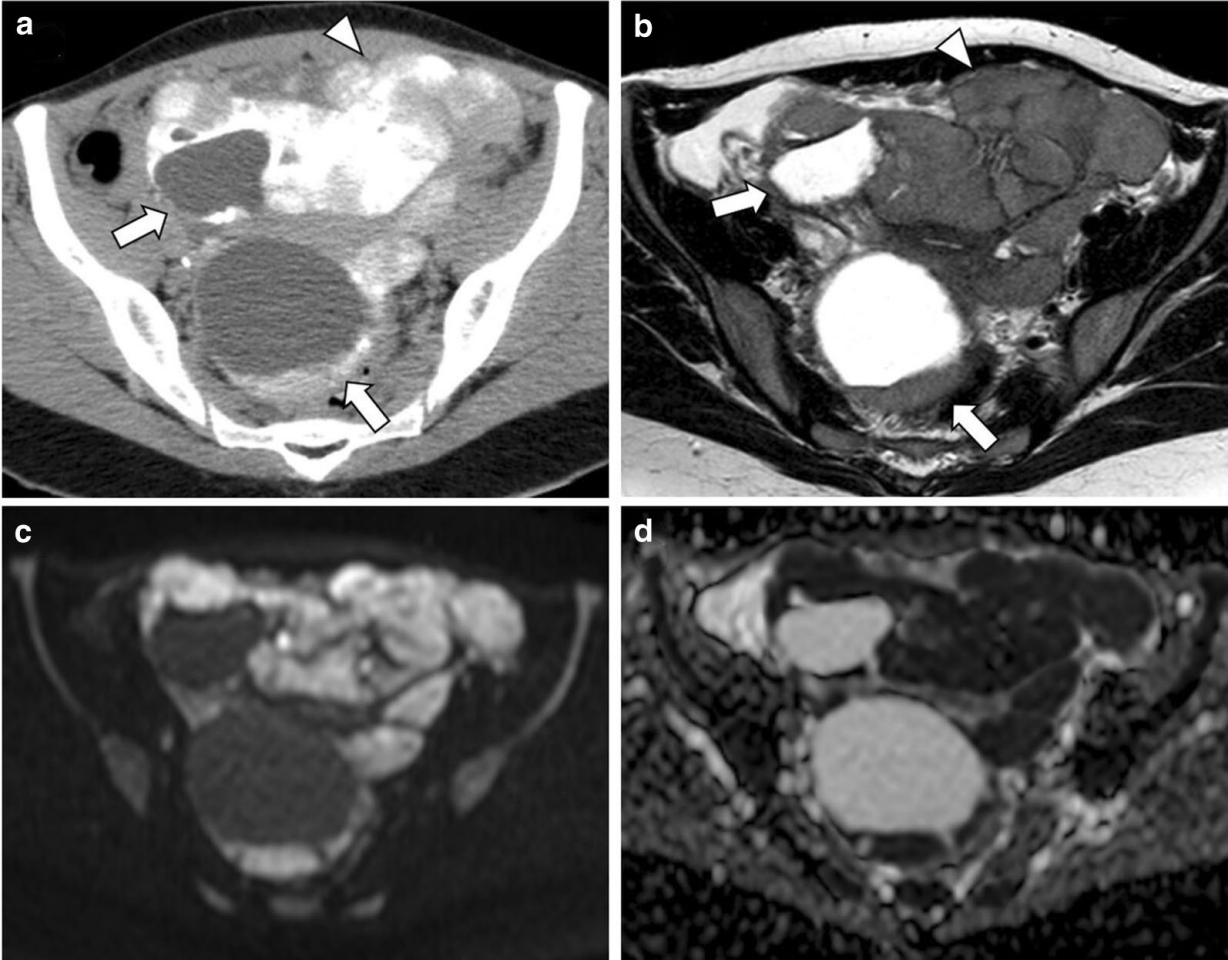
Bilateral ovary LGSC with multiple peritoneal metastases in a 22-year-old female. Axial unenhanced CT image (**a**) and axial T2-weighted MR image (**b**) demonstrate a complex bilateral mixed ovarian tumour, with solid parietal components (arrows) and multiple pelvic solid metastases with exuberant calcified psammoma bodies (arrowheads). Axial *b*-1000 s/mm^2^ diffusion-weighted image (**c**) and ADC map (**d**) show diffusion restriction in parietal nodules and peritoneal metastases

**Fig. 5:**
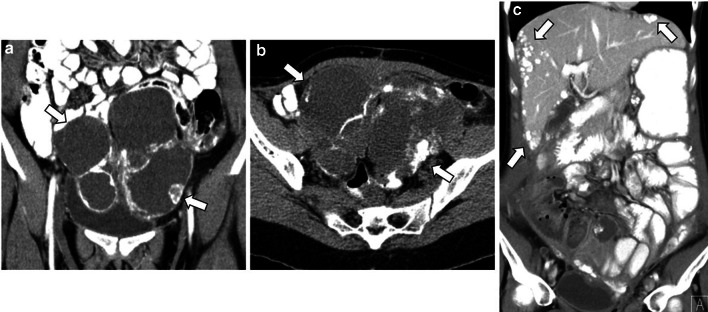
Bilateral LGSC in a 28-year-old female. Coronal and axial enhanced CT images (**a**, **b**) display a bilateral multicystic tumour with thick septa and parietal nodules with multiple psammoma bodies (arrows). Enhanced CT image (**c**), 3 years after cytoreductive surgery, reveals peritoneal metastases on the hepatic surface and in subhepatic space with exuberant calcified psammoma bodies (arrows)

**Fig. 6:**
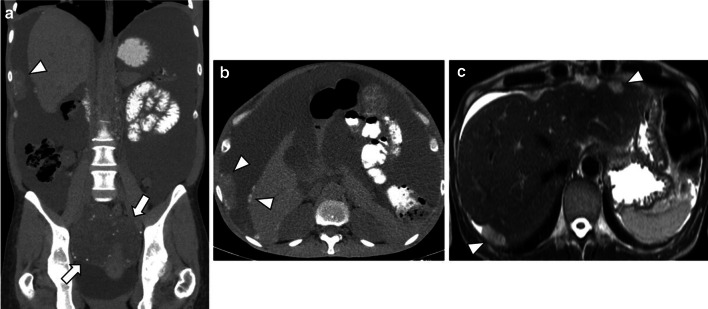
Bilateral LGSC with foci of SBT with peritoneal metastases in a 48-year-old female. Coronal and axial unenhanced CT images (**a**, **b**) display a pelvic bilateral cystic tumour (arrows) with several calcified psammoma bodies and calcified peritoneal metastases, namely in the hepatic surface and in the right hypochondrium peritoneum (arrowheads). Severe ascites is also seen, which is a rare manifestation in this type of tumour. Axial T2-weighted MR image (**c**) demonstrates metastases adherent to the liver surface (arrowheads)

**Fig. 7:**
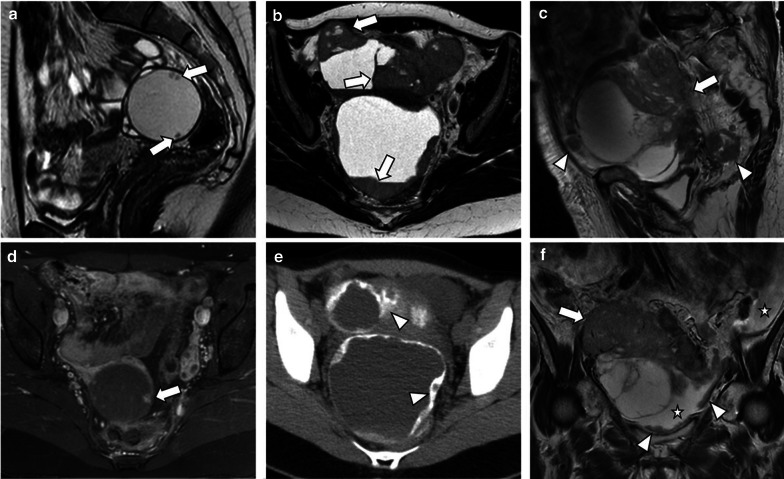
Three cases demonstrating the main radiological features of SBT, LGST and HGST. Sagittal T2-weighted image (**a**) and axial fat saturation T1-weighted image after gadolinium administration (**d**) reveal a right unilocular cystic tumour with well-defined margins and small enhancing papillary projections (arrows). Histologic examination was compatible with SBT. Axial T2–weighted MR image (**b**) and axial unenhanced CT image (**e**) show a bilateral mixed LGST with solid parietal components (arrows) and calcified psammoma bodies (arrowheads). Sagittal and coronal T2-weighted images (**c** and **f**) demonstrate a bilateral mixed HGST with irregular contours and exuberant solid components (arrows). Several abdominal (not shown) and pelvic peritoneal metastases (arrowheads) and ascites (stars) were noted

LGSC can typically appears on CT as a large, complex, cystic mass with well-marginated septa, papillary projections, and solid components that may be found unilaterally or bilaterally [1, 2, 10, 17] (Figs. 4, 5, 6, 7). The number and complexity of serous tumours on solid tissue correlate with malignancy risk [17].

LGSC is characterised by delayed metastatic dissemination, usually through nodular peritoneal metastases throughout the abdomen [2] (Figs. 4, 6).

The classic psammoma bodies, calcified extracellular bodies, can occur within serous tumours or peritoneal metastases, especially in LGSCs (90% of cases) [4, 10–12, 17] (Figs. 5, 6, 7). These tiny calcifications are detected in 30% of tumours at histology but only in 12% of cases at CT [2]. Several authors endorse the use of non-contrast CT and intestinal opacification with water to differentiate tiny calcifications from intestinal loops, particularly in LGSC [1, 13].

Therefore, LGSCs must be distinguished from adnexal lesions that also course with calcifications, such as leiomyomas, Brenner tumours, fibromas, and teratomas [1, 4, 10].

Leiomyomas may arise with popcorn, peripheral, or dense calcifications, but feeding vessels arising from the uterus are usually seen [1, 4, 18, 19].

Brenner tumours can have calcifications similar to those of LGSCs, usually amorphous and central; however, distant metastases are generally not present, since in the vast majority of cases, these tumours are benign [1, 2, 4].

Focal calcifications have been described in less than 10% of fibromas, and the presence of fat distinguishes a teratoma from an LGSC [1, 4, 20].

Peritoneal metastases, nodal calcifications, papillary projections in cystic lesions, and the presence of necrosis are findings that suggest malignancy and help to distinguish malignant serous tumours from benign lesions [1, 4, 17]. Ascites is also rarely identified in LGSC, whereas HGSC typically presents abundant ascites and diffuse peritoneal metastases (Figs. 6, 7) [1, 4, 8].

Dual-energy CT (DECT) is a promising technique that permits the acquisition of variable data by analysing the attenuation of materials at different energy levels in just one CT acquisition [21, 22].

Iodine, a component widely used in CT contrast, is highlighted when low kiloelectron volt (KeV) values are used. This property enables distinguishing structures with this compound from others [21, 22].

Post-processing software also allows additional information to be obtained. One example is the selective removal of certain types of material from the image, that enables to create virtually unenhanced images without iodine, among other uses [22].

Benveniste et al. believe that DECT can be an essential tool in malignant adnexal lesion characterisation since their complexity stands out using iodinated contrast and low KeV values technique [22].

Calcified peritoneal metastases, frequently seen in LGSC, can also be better depicted on low KeV values and water-enhanced images when iodine-based oral contrast is used. In this scenario, intravenous and oral contrasts are removed with post-processing software resulting in virtual unenhanced images and allowing better conspicuity to detect calcified metastases, especially those in the bowel wall [22].

Although this preliminary data indicate that DECT has diagnostic potential in evaluating gynaecological cancer, further studies are needed in this area [21, 22].

### Positron emission tomography (PET)/CT

**Fig. 8:**
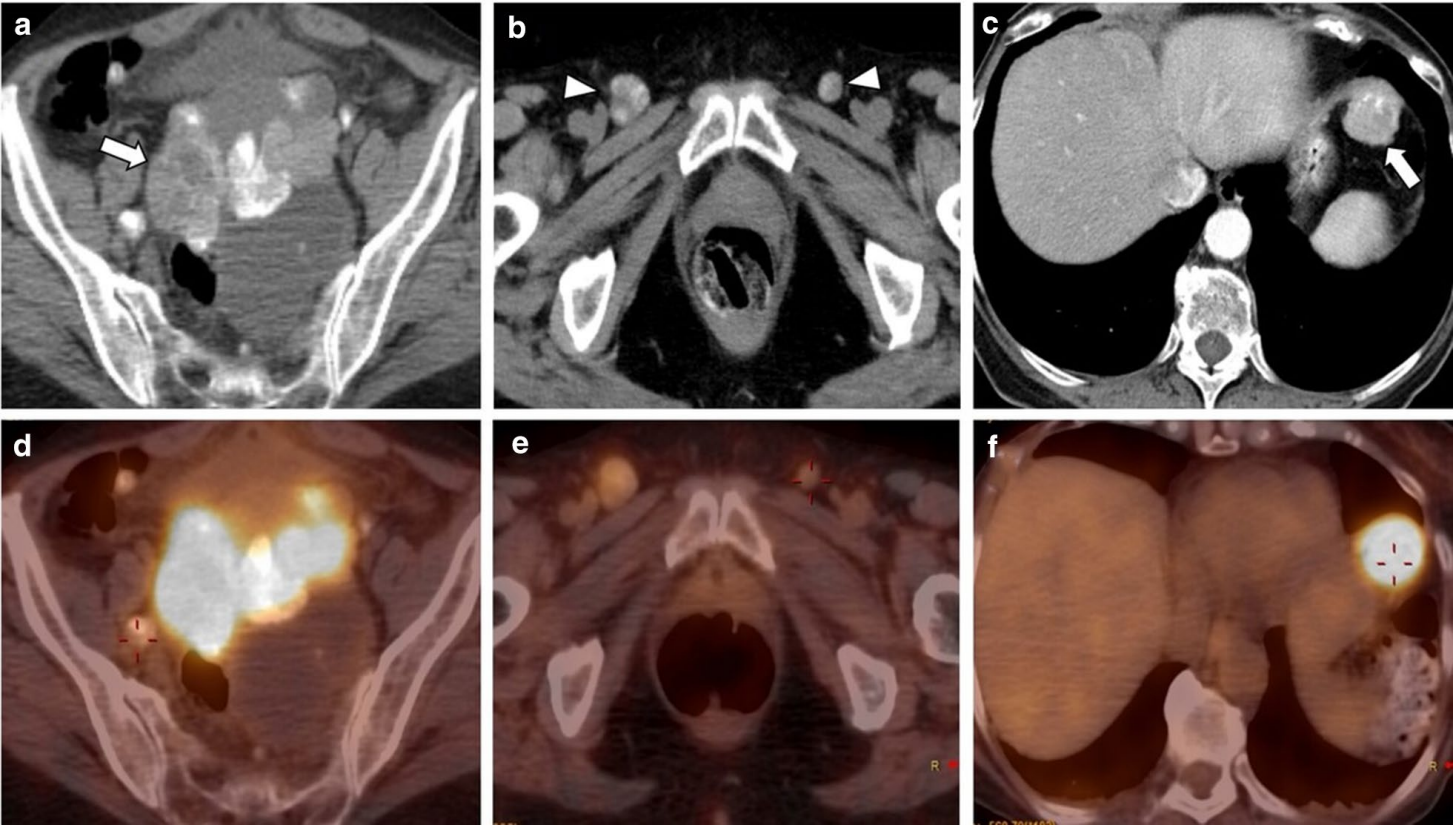
Local recurrence of LGSC with peritoneal and lymph node metastases in a 74-year-old female. Axial unenhanced CT images display a large, heterogeneous, mixed tumour in the vaginal dome (arrow) (**a**) with bilateral inguinal lymph node metastases (arrowheads) (**b**), both with calcified psammoma bodies. Axial enhanced CT image reveals a peritoneal metastasis in the left hypochondrium peritoneum (arrow) (**c**). FDG-PET/CT images show a hypermetabolic pelvic lesion (**d**), bilateral inguinal lymphadenopathies (**e**) and a left upper hypochondrium peritoneal lesion (**f**), suggestive of malignancy

Fluoro-2-deoxy-d-glucose (FDG) PET/CT has a limited role in the primary diagnosis of adnexal masses since false-negative findings have been detected with borderline tumours, mucinous tumours, and other low-grade types of tumours. False-positive results have also been reported with bowel loops, follicular cysts, corpus luteum cysts, and in some benign ovarian tumours [1, 2, 10, 15]. Yet, despite this, FDG-PET/CT can help diagnose and stage advanced disease (stage IV disease), specially when CT is indeterminate [1, 13, 15]. FDG-PET/CT metabolic activity provides disease detection in small metastases or lymph nodes, which can be difficult to characterise only with CT [13, 15] (Fig. 8).

Recently, FDG-PET/CT has revealed similar or higher sensitivity (95–97%) and specificity (80–100%) than CT or PET alone to detect recurrent or residual disease [1, 15, 23].

The identification of metabolic activity in infracentimetric metastases and the detection of disease between intestinal loops, especially after surgery, are recognised limitations of PET [1, 15]. Despite this, the anatomical resolution and metabolic activity of FDG-PET/CT outperform those of CT and MRI in detecting lymph nodes recurrent disease and unresectable sites [1, 15].

### Magnetic resonance imaging

MRI is the modality of choice to characterise indeterminate or large adnexal masses detected on ultrasound or CT, with high sensitivity (83%), specificity (84%), and diagnostic accuracy (83%) [1, 8].

The imaging findings used to predict malignancy and the ancillary findings that improve diagnostic confidence are listed in Table 2 [2, 24]. However, each of these criteria alone does not have sufficient specificity to diagnose ovarian cancer [2, 24].

**Table 2:** Features suggestive of malignancy

**Primary findings**
Lesion size > 4 cm
Wall/septal thickness > 3 mm
Papillary projections
Lobulated mass
Necrosis
Solid and cystic architecture
Type 3 time-intensity curve
**Ancillary findings**
Lymph node enlargement
Peritoneal lesions
Ascites

Primary and ancillary findings used for prediction of malignancy based on the literature review [2, 6]

**Fig. 9:**
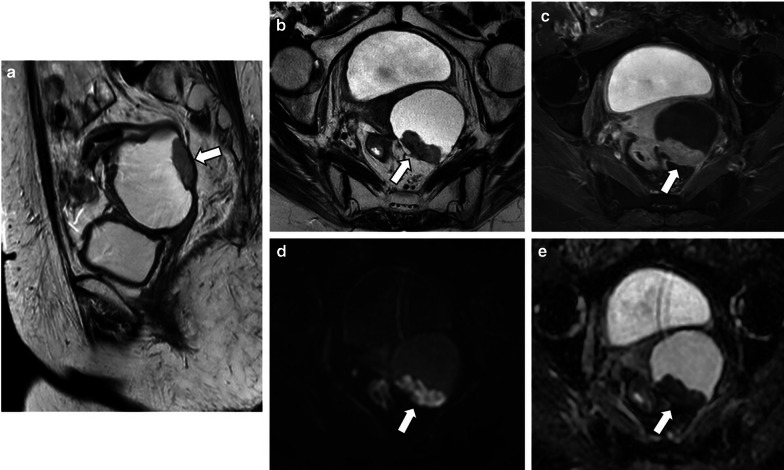
Bilateral LGSC in a 58-year-old female. Sagittal and axial T2-weighted MR images (**a**, **b**) and axial T1-weighted MR image with fat saturation, after gadolinium administration (**c**) demonstrate a left cystic adnexal tumour with solid parietal components (arrows). Axial *b*-1000 s/mm^2^ diffusion-weighted image (**d**) and ADC map (**e**) show diffusion restriction in the parietal nodule (arrows)

Malignant serous tumours are less frequently cystic compared to borderline (respectively 25% and 44%). They tend to be complex mixed lesions with indistinct solid-cystic interfaces [8] (Figs. 1, 3, 4, 7, 9).

Although diffusion-weighted imaging (DWI) characteristics of benign and malignant adnexal lesions may overlap, DWI can be helpful in excluding malignancy when low signal intensity is identified on high *b*-value images [2, 6, 8]. It is also known that the solid components of LGSCs present lower T2 signal intensity and lower ADC values than SBTs [2, 5] (Fig. 1).

The evaluation of MRI contrast enhanced sequences is an essential step in tumour characterisation. It allows a more detailed assessment of the papillary projections seen on serous tumours and the characterisation of their vascularisation patterns [2]. It also confirms or excludes the presence of necrosis [2].

The characterisation of the solid components of complex adnexal masses using a semiquantitative multiphase-dynamic contrast-enhanced MRI technique has shown to discriminate benign, borderline, and malignant ovarian tumours [2, 6, 8, 25]. Solid components that show a rapid and high enhancement level are associated with a very high malignancy likelihood [25].

**Fig. 10:**
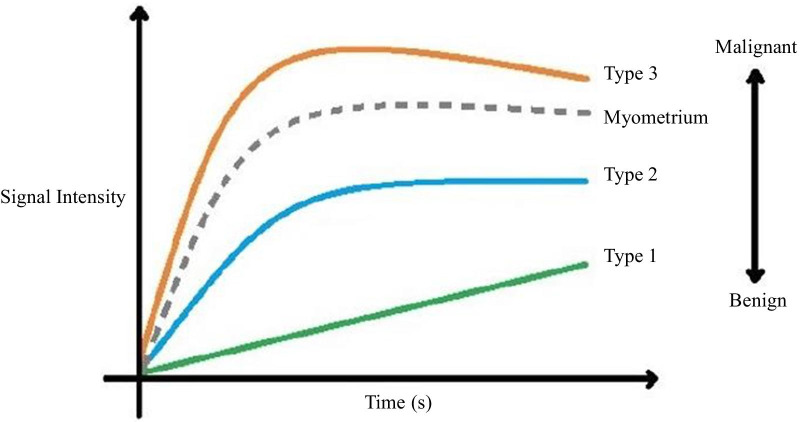
Time-intensity curves. Time-intensity curves compare the enhancement patterns of the ovarian tumour's solid aspect with the outer myometrium

**Fig. 11:**
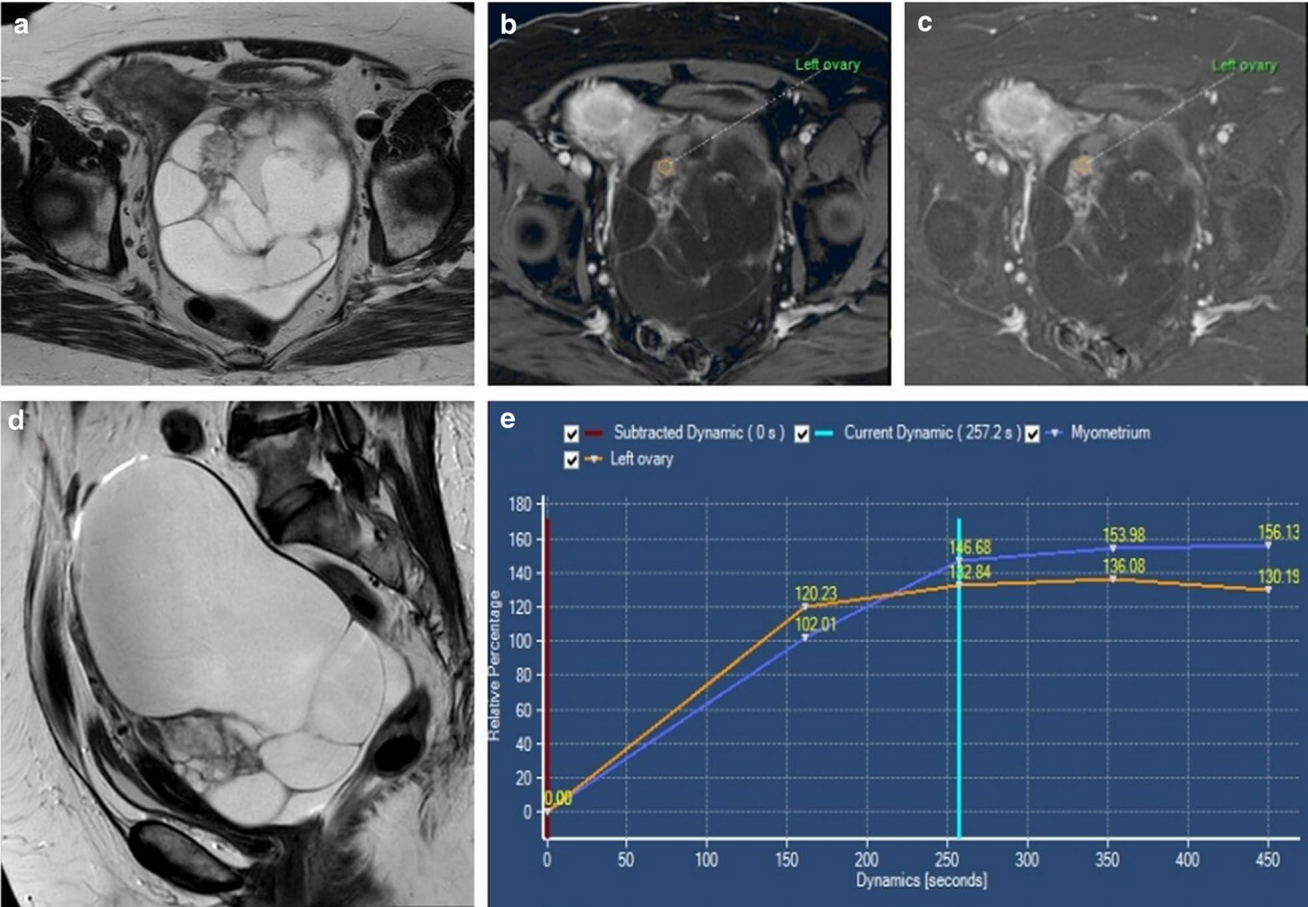
LGSC of the left ovary in a 61-year-old female. Axial and sagittal T2-weighted MR images (**a**, **d**), axial T1-weighted MR image with fat saturation, after intravenous gadolinium administration (**b**) and post-contrast subtraction (**c**) demonstrate a large multicystic left ovary tumour, with some thick septations and solid parietal nodules. The larger nodule displays a type 3 contrast enhancement curve (**e**), which is commonly found in malignant epithelial ovarian tumours. It shows an initial contrast uptake higher than the myometrial uptake, followed by washout

This technique identifies three types of enhancement curves by comparing the solid enhancement pattern of the lesion with myometrial enhancement [2, 6, 8, 26, 27] (Fig. 10). Type II curves (early and moderate uptake of gadolinium, not exceeding the myometrial signal, followed by a plateau) are typical of borderline tumours, whereas type III curves (avid and early contrast uptake, more accentuated than the myometrium’s, followed by washout) are more commonly seen in malignant epithelial ovarian tumours, such as LGSCs (Fig. 11). Type I curves (gradual uptake of contrast) are characteristic of benign lesions [2, 6, 8, 27].

These enhancement patterns are also included in the MRI ADNEX scoring system, which classifies the probability of malignancy of complex adnexal lesions [1, 2, 26, 28].

Recently, a multicentre study validated the Ovarian-Adnexal Reporting and Data System (O-RADS) MRI risk stratification scoring system, which allows the standardisation of risk stratification and provides indications for follow-up of adnexal masses using MRI and the O-RADS ultrasound score. This score showed high sensitivity and specificity (93% and 91%) to diagnose malignant lesions amongst indeterminate masses detected by ultrasound [28–30].

MRI has gained value as an alternative technique for staging ovarian cancer when DWI is used with standard sequences [13]. MRI is preferred over CT if there are contraindications to iodine contrast and in pregnant and young women [1, 2, 13].

MRI is also helpful in evaluating treatment response and in excluding recurrent disease, as dynamic contrast-enhanced (DCE) MRI allows detection of residual and recurrent peritoneal disease with a sensitivity of 90% and specificity of 88% [31].

Generally, ovarian cancer peritoneal metastases, such as those from LGSC, demonstrate high signal intensity on DWI and low signal intensity on ADC and are best evaluated 5–10 min after paramagnetic contrast administration [31] (Figs. 3, 4).

DWI also showed high sensitivity in detecting small peritoneal metastases, mainly in the pouch of Douglas and the left upper quadrant.

## Conclusion

LGSC is a rare subtype of epithelial serous tumour. LGSC and HGSC have a distinct histogenesis, clinical behaviour, sensitivity to chemotherapy, and prognosis; therefore, preoperative discrimination between LGSC and other serous tumours is fundamental to guide patient care and treatment strategies.

Although differentiation between these subtypes is only entirely possible histologically, imaging can provide clues that may suggest the diagnosis of LGSC.

LGSCs can appear as a solid, mixed solid cystic, or complex cystic adnexal mass. Classic psammoma bodies are frequent in this type of tumour and can occur within the adnexal mass, lymph nodes and peritoneal metastases. Moreover, MRI evaluation of lesion enhancement pattern can also provide important tips to discriminate benign, borderline, and malignant ovarian tumours.

As such, in addition to be aware of the most frequent radiological findings of LGSC, radiologists must also be familiarised with the pathology, biology, and characteristic markers of this tumour to optimise the interpretation of images and provide adequate management and timely treatment to these women.

## Data Availability

Data sharing is not applicable to this article as no datasets were generated or analysed during the current study.

## Abbreviations

ADNEX: Assessment of Different NEoplasias in the adnexa
CT: Computed tomography
DCE: Dynamic contrast-enhanced
DECT: Dual-energy CT
DWI: Diffusion-weighted imaging
FDG: Fluoro-2-deoxy-d-glucose
HGSC: High-grade serous carcinoma
IOTA: International Ovarian Tumour Analysis
KeV: Kiloelectron volt
LGSC: Low-grade serous carcinoma
MRI: Magnetic resonance imaging
O-RADS: Ovarian-Adnexal Reporting and Data System
PET/CT: Positron emission tomography/Computed tomography
SBT: Serous borderline tumour
WHO: World Health Organization
